# Clinical and genetic analysis of a female child with duplications at 7p22.3p22.1 and Xp22.31p21.1: A case report

**DOI:** 10.1097/MD.0000000000041452

**Published:** 2025-02-07

**Authors:** Chenchen Bu, Quzhen Zha Xi, Ying Deng

**Affiliations:** a Department of Pediatrics, Tibet Maternal and Child Health Hospital, Lhasa, China; b Department of Pediatrics, West China Second University Hospital, Sichuan University, Chengdu, China; c Key Laboratory of Birth Defects and Related Disease of Women and Children, Ministry of Education, Sichuan University, Chengdu, China.

**Keywords:** 7p22.3p22.1 duplication, case report, intellectual disability, whole exome sequencing, Xp22.31p21.1 duplication

## Abstract

**Rationale:**

Intellectual disability (ID) is a neurodevelopmental disorder with diverse etiologies. Chromosomal duplications are recognized as a common cause of ID, with manifestations typically milder than those associated with deletions. Duplications involving the short arm of chromosome 7 or the X chromosome have been linked to ID. Limited data exist on duplications at 7p22.1p22.3 and Xp22.31p21.1, leaving their clinical significance largely unexplored. This case report aims to expand the phenotype and genetics spectrum of 7p22 duplication syndrome and X-linked ID. Special attention should be paid to closely monitoring the pubertal progression of such patients.

**Patient concerns:**

A 9-year-10-month-old female was admitted to our hospital due to distinctive dysmorphic features and ID.

**Diagnoses:**

Upon examination, features of craniofacial dysmorphism were observed, including micrognathia, a prominent lower lip, a thin upper lip, a flat nasal bridge, and hypertelorism. The child’s pubertal development is progressing extremely rapidly; at under 10 years old, the breasts have already advanced to Tanner Stage 4. Her height was within the median range, but her bone age was advanced (12.1 years). Her full-scale intelligence quotient, assessed using the Wechsler Intelligence Scale for Children: 4th edition, was 41.

**Interventions:**

Whole exome sequencing identified a de novo duplication spanning overlapping regions at Xp21-22 and 7p22. This duplication encompasses several genes implicated in ID, including Duchenne muscular dystrophy, Aristaless-related homeobox, interleukin-1 receptor accessory protein like 1, adaptor-related protein complex 1 subunit sigma 2, and cyclin-dependent kinase-like 5.

**Outcomes:**

A novel duplication in the Xp and 7p of a Chinese female child diagnosed with ID and dysmorphic features has been studied by whole exome sequencing analysis. The novel duplications were a large duplication located in the 7p22.1 to p22.3 region, spanning 12.07 Mb, and a large duplication located in the Xp22.31 to p21.1 region, spanning 24.7 Mb.

**Lessons:**

Our study underscores the importance of comprehensive clinical and genetic evaluation in individuals with duplication on the X chromosome and the terminal region of chromosome 7’s short arm. We highlight the need for monitoring these patients for growth and sexual development issues. Our findings also suggest that chromosomal duplication can lead to severe clinical manifestations, emphasizing the critical role of genetic assessment in managing such cases.

## 1. Introduction

Intellectual disability (ID) is a neurodevelopmental disorder characterized by impairments in intellectual and adaptive skills, affecting at least 1 of the 3 adaptive domains (conceptual, social, and practical) with varying degrees of severity.^[1]^ It is prevalent in the general population, with an incidence of approximately 1% to 2%.^[2]^ The causes of ID are numerous, including conditions that disrupt brain development and function. Among the known causes of ID, the majority are genetic abnormalities. A specific genetic cause can be identified in more than 50% of individuals with ID referred for evaluation.^[3]^ Partial duplication of the short arm of chromosome 7 is a rare genetic disorder with undetermined prevalence and clinical significance. There are more than 60 reports published on the topic of 7p chromosome replication. The size of the duplicated segments varies among patients.^[4–6]^ The phenotype spectrum also varies accordingly. The clinical manifestations include high-frequency hypotonia, ID, autism spectrum disorder, cardiovascular and skeletal abnormalities, as well as certain craniofacial malformations. According to previous cases, males with 7p22 duplications have presented with cryptorchidism.^[7]^ However, there is limited reporting in the literature concerning the impact of the 7p22 duplication on the puberty and sexual development of female patients.

It has been reported that mutations leading to X-linked intellectual disability (XLID) are present in over 100 genes, accounting for 5% to 10% of male ID cases. X-linked disorders are highly heterogeneous, presenting in either a syndromic or nonsyndromic form.^[1]^ Among affected XY males, the only consistent phenotypic features appear to be ID and dysmorphic facial features. Other phenotypes are highly diverse depending on the size and location of the duplication on the short arm of the X chromosome. Some reported clinical features include obesity, cleft palate, epilepsy, short stature, sex reversal, and skeletal abnormalities.^[8]^ In female patients with X chromosome duplication, phenotype prediction is more challenging. The normal or mild phenotype in females may be due to the selective inactivation of the abnormal X chromosome. In the majority of female patients with X chromosome rearrangements, it has been found that the X chromosome with the rearrangement is preferentially inactivated.^[9]^ The majority of females do not exhibit abnormal phenotypes or are mildly affected compared to males, often showing characteristics of Turner-like syndrome and normal intelligence.^[10]^ Thorson et al^[8]^ reported a 4 1/2-year-old female who presented with mild hypotonia and moderate expressive language delay. The array comparative genomic hybridization indicated that cytogenetic analysis detected a duplication affecting the segment between Xp21.2 and Xp22.2 on the X chromosome. The duplicated region is 14.5 Mb in size and contains over 80 known genes. The patient has a relatively normal height (height at 25th centile). Therefore, the clinical manifestations of X chromosome duplication are heterogeneous, with a wide range of symptoms, from asymptomatic to severe phenotypes, especially for females. Due to the limited number of reported cases, a comprehensive clinical phenotype remains elusive, and there are currently no case reports of female patients with both X chromosome short arm duplication and 7th chromosome short arm duplication.

The increasing use of next-generation DNA sequencing technologies, such as whole exome sequencing (WES), has led to the discovery of more genes associated with both syndromic and nonsyndromic forms of ID. In the present study, we report a case of a 9-year-10-month-old female referred for developmental delay and dysmorphic features, who was found to have an Xp21.1p22.31 duplication and 7p22.3p22.1. The detailed clinical and molecular characteristics of the patient enable us to better correlate their phenotypes with genotypes, allowing for better delineation of the clinical presentation of this chromosomal duplication syndrome and further determination of the chromosomal critical region.

## 2. Case presentation

### 2.1. Patient information

A 9-year-10-month-old female child was referred to our clinic for further clinical and genetic evaluation, as the girl had dysmorphic features and developmental delay. She was the second child of healthy nonconsanguineous parents (mother 27 years; father 28 years), born at term from an uneventful pregnancy. Her birth weight was normal, and no perinatal suffering was reported (appearence-pulse-grimace-activity-respiration score 10-10-10). There were no reported developmental disabilities or schizophrenia in her family history, and her parents and brother are healthy. No dysmorphic features were noted in her parents or brother. Developmentally, she achieved the milestone of sitting at 1.5 years old, standing at 3 years old, and walking alone at 4 years old. Expressive speech delay was observed during the second year of life. She began to speak several words at 4 years old. The patient had no intracranial infection history and no history of taking special medications. Up to now, the patient is currently in the third grade and is struggling academically, unable to keep up with the teacher’s pace in class. She can only engage in simple communication and has limited comprehension abilities.

### 2.2. Physical examination

**Figure 1.**
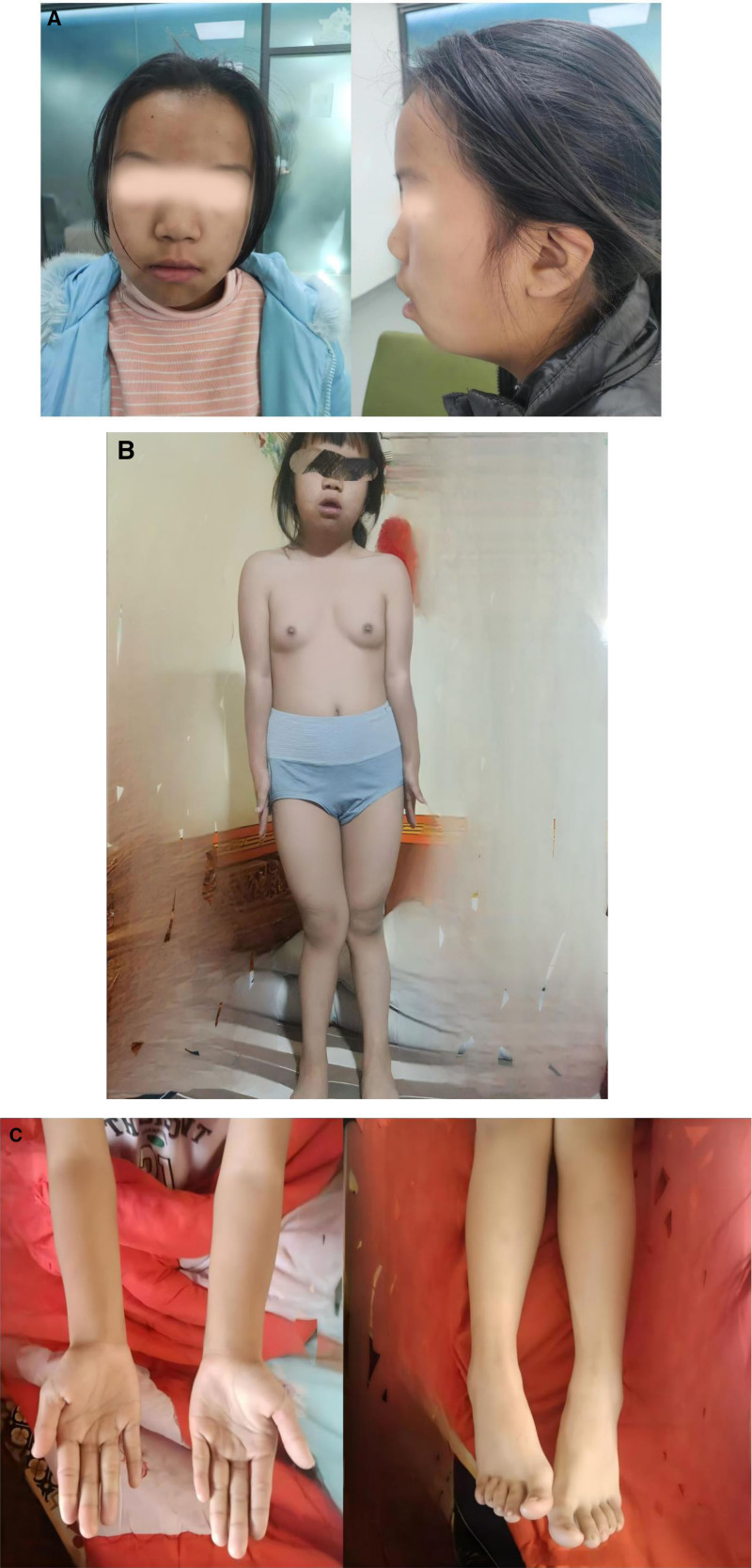
(A) Facial photograph of the proband (frontal view and lateral view) showing dysmorphic features such as micrognathia, a prominent lower lip, a thin upper lip, a flat nasal bridge, and hypertelorism. (B) The full-body photo of the patient showed no skeletal deformities and scoliosis. Her breasts had already advanced to Tanner Stage 4. (C) Features of the patient’s hands and feet.

Upon examination, her hearing test results were normal, although an eye examination indicated misalignment of the eyes. Features of craniofacial dysmorphism were observed, including micrognathia, a prominent lower lip, a thin upper lip, a flat nasal bridge, and hypertelorism (Fig. 1A). There were no abnormalities in the spinal physical examination, and no scoliosis was found. The child could complete simple instructions, and there were no abnormalities in walking posture and climbing stairs (Fig. 1B). The child’s pubertal development is progressing extremely rapidly; at under 10 years old, the breasts have already advanced to Tanner Stage 4 (Fig. 1C). The physical examination showed that she had a normal stature with a body length of 136.1 cm (34th centile). The weight was 37 kg (86th centile), and the head circumference was 54 cm.

### 2.3. Diagnostic testing

**Figure 2.**
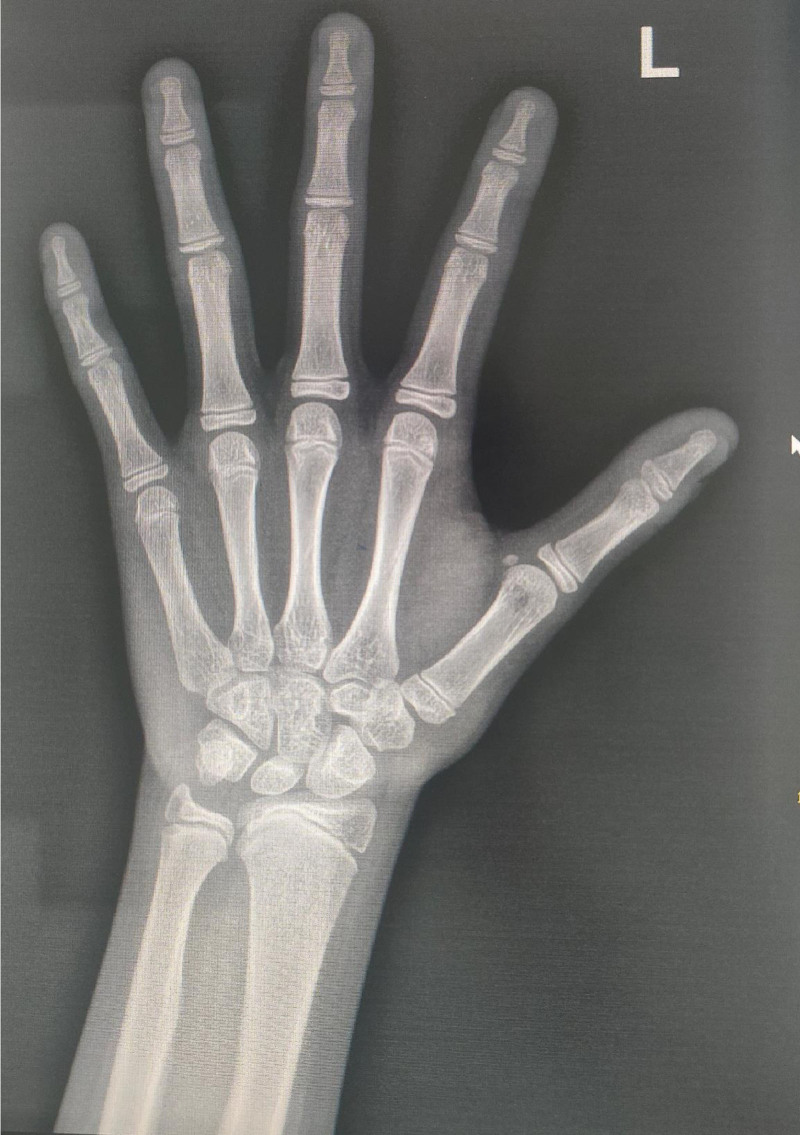
The bone age was precocious, with a bone age of 12.1 years.

**Figure 3.**
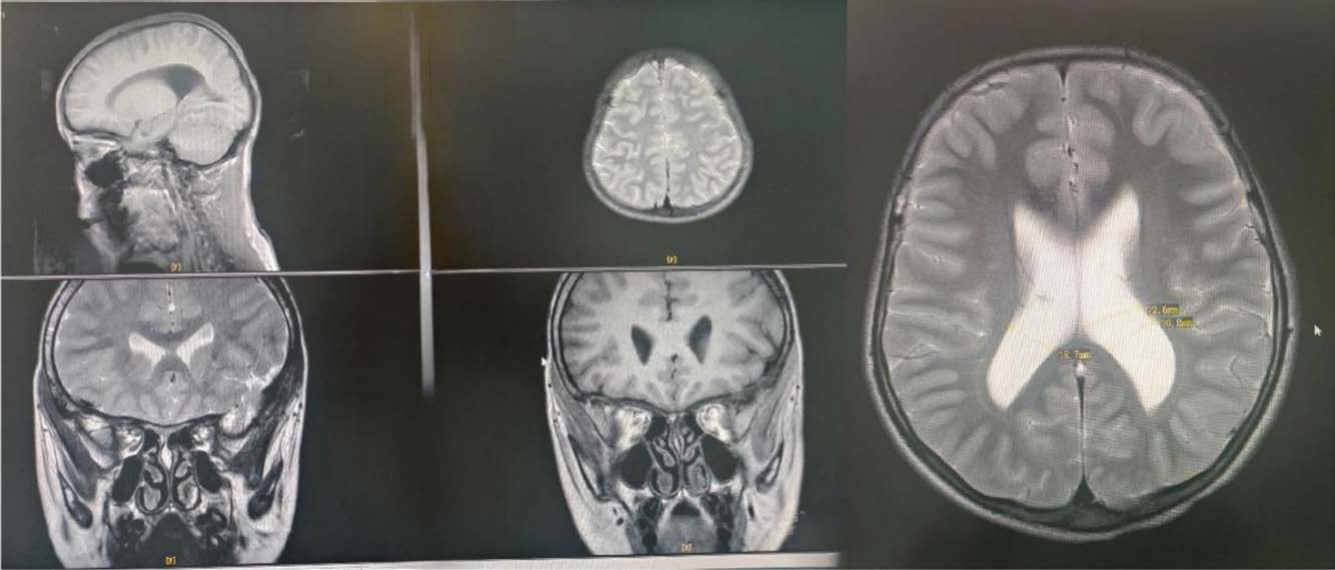
Magnetic resonance imaging of the brain revealed widening of bilateral lateral ventricles (left ventricle 2 cm and right ventricle 1.8 cm).

The child’s bone age is precocious, with a bone age of 12.1 years (Fig. 2). Then, she underwent a cognitive level test using the Wechsler Intelligence Scale for Children: 4th edition, which revealed that her full-scale intelligence quotient was 41, Verbal Comprehension Index was 45, Perceptual Reasoning Index was 50, Working Memory Index was 45, and Processing Speed Index was 45. Echocardiography, abdominal ultrasound, and thyroid function studies were unremarkable. Magnetic resonance imaging of the brain revealed widening of bilateral lateral ventricles (left ventricle 2 cm, right ventricle 1.8 cm) (Fig. 3). WES was performed in the patient and both parents. Briefly, genomic DNA extraction from peripheral blood leukocytes was conducted using the Lab-Aid DNA kit. Exons were captured using the Agilent SureSelect Human All ExonV5 Kit (Agilent) based on the manufacturer protocols. Sequencing was performed on an Illumina NovaSeq 6000 platform with 150 bp paired-end reads. Reads were aligned to the human genome assembly GRCh37 and analyzed for sequence variants using a custom-developed analysis tool.

### 2.4. Outcomes, therapeutic intervention, and follow-up

**Figure 4.**
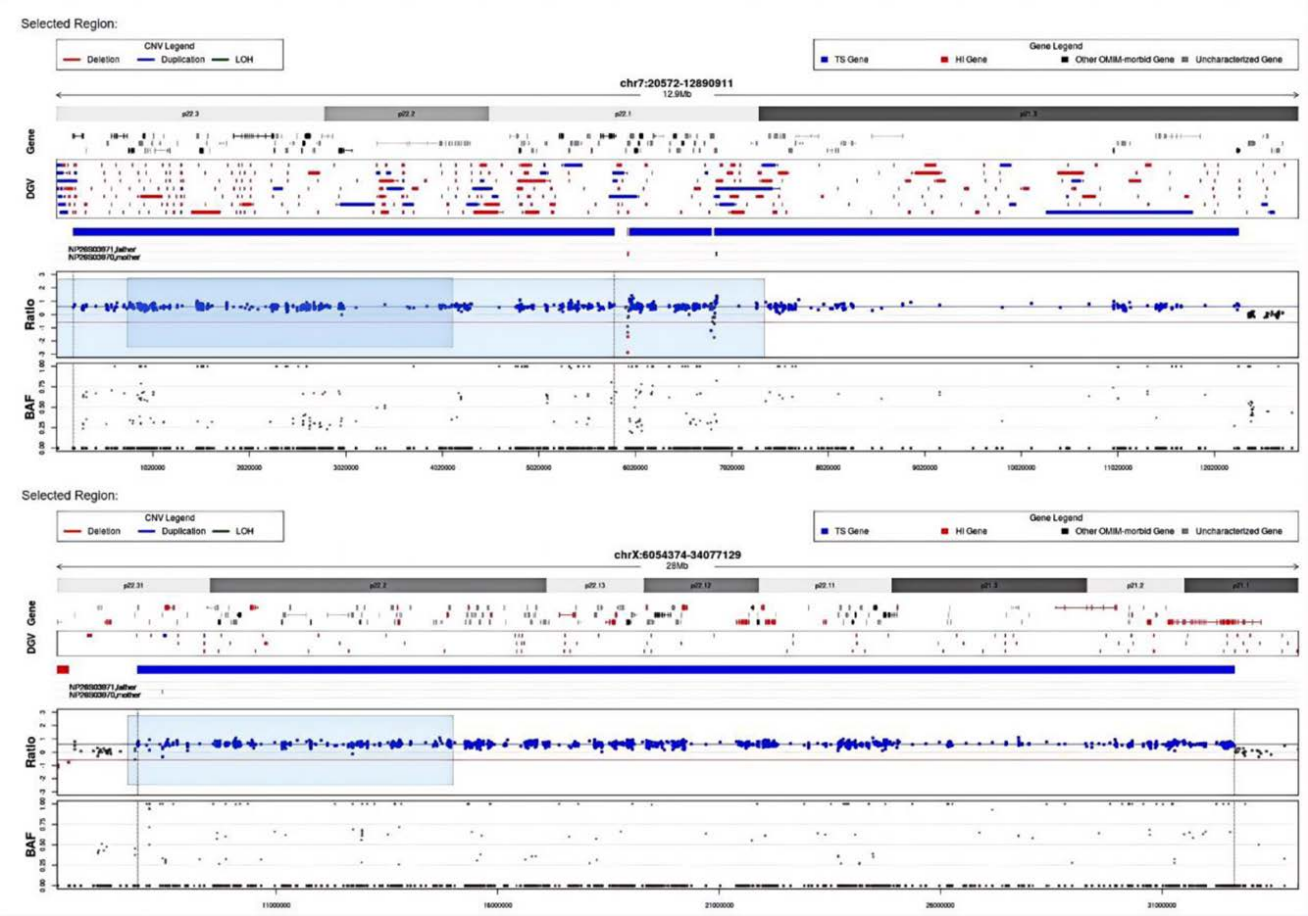
Whole exome sequencing results of the proband and parents. It showed a large de vono duplication located in the 7p22.1p22.3 and Xp22.31p21.1 regions. BAF = B-allele frequency, CNV = copy number variation, DGV = database of genomic variants, HI = homozygous indel, LOH = loss of heterozygosity, OMIM = Online Mendelian Inheritance in Man, TS = translocation site.

Our case showed a large duplication located in the 7p22.1p22.3 region spanning 12.07 Mb from position 20572 to position 12890911. In addition, another large duplication spanning 24.7 Mb from position 6054374 to position 34077129 was also detected in the Xp22.31p21.1 region. Both parents were confirmed to not be carriers of the same duplication (Fig. 4). Unfortunately, the parents, due to financial constraints, declined to proceed with array comparative genomic hybridization. It is recommended that the child be enrolled in a special education class to ensure they receive the tailored support they need. Additionally, it is essential that the parents bring the child to the growth and development clinic for a checkup every 6 months, ensuring their progress is closely monitored and any necessary interventions are promptly addressed.

## 3. Discussion

The case presented here involves a girl aged 9 years and 10 months, with a large duplication of 7p and Xp, along with a constellation of global delays, including motor and speech delays, with greater involvement of expressive language, learning difficulties, advanced bone age, and dysmorphic features. WES of our patient revealed a de novo 12.07 Mb dup 7(p22.1p22.3) and 24.7 Mb dup X(p22.31p21.1). The 7p22 duplication syndrome is characterized by developmental delay, ID, and craniofacial dysmorphisms. Cardiac and skeletal abnormalities are often present. According to literature reports, there have been over 60 cases of patients with 7P duplication syndrome.^[4–7,11]^ Caselli et al^[7]^ reported the first case of an adult patient aged 35 years with moderate ID, psychomotor retardation, facial dysmorphism, cryptorchidism, and cardiac malformation, who carried 2 proximal microduplications at 7p22.1, approximately 900 and 150 kb, respectively. The other cases also indicated that male patients with 7p22 duplication syndrome were prone to cryptorchidism.^[11–13]^ The patient described in the current report had central nervous system abnormalities and ID compared to patients in previous reports.^[14,15]^ Unlike the other patients, our proband did not show relative or absolute macrocephaly at the time of evaluation (Table 1). Despite the patient having neurological abnormalities, she only manifested as bilateral ventricular enlargement, rather than hydrocephalus. Although skeletal anomalies seem to be a common clinical finding since they are present in 3 previously described patients,^[7,14,15]^ our patient and the one described by Pebrel-Richard et al^[13]^ do not show skeletal anomalies. An alternative hypothesis is that the absence of skeletal abnormalities in the 2 patients may be due to incomplete penetrance and/or variable expressivity of the relevant genes, a hypothesis that has been proposed for several microduplication syndromes.^[16]^ The incomplete penetrance of the duplicated genes at the terminal end of the short arm of chromosome 7 seems to explain why the case we reported, despite having a large segmental duplication, does not exhibit more severe clinical manifestations than those with smaller segmental duplications. Of course, further research is needed in the future. It is noteworthy that the patient described in this report had no cardiovascular abnormalities, thus resulting in a better prognosis compared to the patient in the previous report (Table 1). Previous case reports have not focused on the progression of puberty in patients. In this study, the child was under 10 years old, but the patient’s bone age had already reached 12.1 years, indicating a significantly advanced bone age, and breast development had progressed to stage 4 of the Tanner stage. Therefore, for the first time, we propose that patients with 7p duplication syndrome need to be monitored for sexual development and the progression of puberty.

**Table 1:** Clinical findings in the present patient and other reported female cases with 7p22 duplications.

	AlFardan et al^[14]^	Preiksaitiene et al^[15]^	Our case
Duplicated region	7p22.1p22.2 (2.4 Mb)	7p22.1 (980 kb)	7p22.1p22.3 (12.07 Mb)
Age at time of report	11 yr 6 mo	14 yr	9 yr 10 mo
Psychomotor delay	+	+	+
Dysmorphic features	Prominent forehead, supraorbital ridges, hypertelorism, low-set ears, small upturned nose with depressed and broad nasal bridge, small upper and prominent lower lips, large incisors, and micrognathia, ocular hypertelorism, macrocephaly	Ocular hypertelorism, downslanting palpebral fissures, low set and/or malformed ears, Low broad nasal bridge, micrognathia and/or retrognathia, abnormal palmar creases macrocephaly	Ocular hypertelorism, micrognathia, a prominent lower lip; thin upper lip, a flat nasal bridge
Skeletal problems	Scoliosis, coax valga, leg-length difference	Scoliosis, short 5th toes, tapering fingers	−
Cardiovascular abnormalities	ASD, PDA	−	−
CNS abnormalities	Dandye–Walker malformation, hydrocephalus	Moderate internal hydrocephalus	Bilateral ventriculomegaly
Genitourinary abnormalities	Vesicoureteral reflux	−	−
Short stature	+	−	−
Other	−	Truncal obesity	0besity, advanced bone age

ASD = atrial septal defect, CNS = central nervous system, PDA = patent ductus arteriosus.

Duplication of part of the X chromosome without concurrent deletions is rare. So far, over 50 instances of Xp replication have been described in the literature.^[8,9,17]^ However, there are very limited examples of molecularly and phenotypically characterized interstitial Xp21-p22 duplications. Our affected individual showed that the large Xp duplication detected by cytogenetic analysis affects the segment between bands Xp21.1 and Xp22.31. The duplicated area for the affected individual encompasses many important genes involved in the central nervous system function (Table 2). Mutations in the Duchenne muscular dystrophy (DMD) gene can lead to 2 types of muscular dystrophy: Becker muscular dystrophy and DMD. Both of these diseases are progressive, characterized by muscle tissue degeneration and replacement with fibrotic tissue, leading to muscle weakness, difficulty or loss of walking, increased creatine kinase levels, and heart failure. One-third of DMD patients also have mental retardation (MR). In both Becker muscular dystrophy and DMD, the prevalence of MR is quite high.^[24,25]^ In this case, the patient did not exhibit typical clinical manifestations associated with DMD, such as muscle atrophy, but only presented with ID, which may be related to a chromosomal duplication. The clinical presentation of patients with duplications is generally milder than those with deletions or mutations.

**Table 2:** Genes in the Xp duplicated regions known to be associated with genetic disorders in humans.

Gene	Protein	Function	Disease association	Reference
*DMD*	Dystrophin	A complex plasmalemmal-cytoskeletal linker protein	Duchenne and Becker muscular dystrophy, X-linked ID	Mehler et al^[18]^
*CDKL5*	Cyclin-dependent kinase-like 5	Protein kinase	X-linked infantile spasm syndrome, Rett syndrome, MR, refractory epilepsy	Hong et al^[19]^
*AP1S2*	Sigma 2 subunit of the adaptor protein 1 complex	Part of the adaptor protein complex at the cytoplasmic face of coated vesicles located at the golgi complex	Loss of function causes MR and Fried syndrome, Pettigrew syndrome	Huo et al^[20]^
*IL1RAPL1*	Interleukin 1 receptor accessory protein-like 1	Possible role in the physiological processes underlying memory and learning ability	Loss of function causes nonsyndromic MR X-linked type 2	Montani et al, Ramos-Brossier et al^[21,22]^
*ARX*	Aristaless-related protein	Essential for cerebral patterning and for the maintenance of specific neuronal subtypes in the cerebral cortex	X-linked lissencephaly with abnormal genitalia, Proud syndrome or mental retardation	Laperuta et al^[23]^

ID = intellectual disability.

The Aristaless-related homeobox (*ARX*) gene encodes the Aristaless-related protein, which is a dual-function homeobox transcription factor crucial for patterning the brain and maintaining specific neuronal subtypes in the cerebral cortex. Its mutations account for 9.5% of X-linked MR families.^[23]^ The duplicated part of the cases we reported is between Xp21 and Xp22, including the *ARX* gene, which is a gene related to brain development. Duplication of the *ARX* gene can cause moderate to severe ID. Interleukin-1 receptor accessory protein like 1 (IL1RAPL1) protein is located at the postsynaptic compartment of excitatory synapses and plays an important role in the formation and stabilization of synapses. Deletions and point mutations in IL1RAPL1 are the cause of nonsyndromic MR X-linked type 2.^[21]^ Studies have shown that the inactivation of the *IL1RAPL1* gene can lead to cognitive deficits in both male and female mutation carriers.^[21,22,26]^ The *IL1RAP* duplication therefore could have contributed to the phenotypic abnormalities in our case.

Cyclin-dependent kinase-like 5 (*CDKL5*) and adaptor-related protein complex 1 subunit sigma 2 (*AP1S2*) are both genes associated with ID. *CDKL5* deficiency disorder (CDD) is a developmental and epileptic encephalopathy associated with infantile-onset seizures, global developmental delay with subsequent intellectual and motor disabilities. The majority of CDD patients develop refractory epilepsy with multiple seizure types.^[19,27]^ CDD patients exhibit severe developmental delay, with only a quarter of girls and a minority of boys achieving independent walking; our case showed severe developmental delay, with the patient only beginning to walk and speak at the age of 4, but without any seizure episodes. The possible reason is incomplete penetrance of the gene, as patients with gene duplications generally have milder symptoms compared to those with gene mutations or deletions. *AP1S2* is a subunit of AP1 that is crucial for the reformation of the synaptic vesicle. Variants in the *AP1S2* gene have been reported to cause a rare neurodevelopmental disorder, Pettigrew syndrome, characterized by delayed walking, abnormal speech, mild-to-severe XLID, and abnormal brain and behavioral features. Zhu et al^[20]^ reported the second case in the Chinese family and the eleventh variant found in *AP1S2*-related XLID. Here, we reported the 3rd in the Chinese family case with XLID associated with mutations in the *AP1S2* gene. The clinical features of our patient were consistent with previous reports.^[20,28]^ Since the *AP1S2* gene mutation was identified as the cause of PGS in 2006, only 11 *AP1S2* mutations have been found in literature, including 9 point mutations and a microdeletion and a duplication. Our research focused on the second duplication in AP1S2.

The strengths of this article lie in the utilization of WES for the etiological diagnosis of a case with distinctive facial features and ID, followed by Sanger sequencing validation. WES has increasingly been applied to the genetic diagnosis of rare diseases. We also conducted a comprehensive literature review, first proposing that the progression of puberty should be monitored in cases involving 7p22 and Xp terminal duplication, as evidenced by accelerated pubertal progression and predicted compromised adult height in our patient.

The limitations are as follows: first, the absence of array comparative genomic hybridization for an accurate description of chromosomal rearrangements; and second, the radiographs of the skull, spine, and hands were not obtained because the parents refused to have them taken. For pediatric patients presenting with craniofacial deformities, radiographic imaging of the skull, spine, and extremities is an essential examination.

## 4. Conclusions

In conclusion, our study shows that WES is a useful tool to screen for ID, which can lead to a molecular diagnosis via additional molecular testing and research. By combining careful clinical analysis, literature, and further genetic characterization, we report novel duplications on chromosome X implicated in X-linked ID, and 7P22 duplication syndrome. Our report expands the phenotype and genetics spectrum of 7p22 duplication syndrome and XLID. For female patients with a combined 7p22 duplication syndrome and XLID, it is important to closely monitor the progression of puberty. Future research will still require in vivo and in vitro experiments to explore the pathogenic mechanisms underlying the multi-system abnormalities caused by the 7p22 and Xp terminal duplication.

## Acknowledgements

The authors would like to thank the family of the proband for their cooperation with this study.

## Author contributions

**Formal analysis:** Chenchen Bu, Quzhen Zha Xi.

**Investigation:** Chenchen Bu, Quzhen Zha Xi.

**Methodology:** Quzhen Zha Xi.

**Writing – original draft:** Ying Deng, Chenchen Bu.

**Writing – review & editing:** Ying Deng.
